# Evaluation of Proteomic and Lipidomic Changes in *Aeromonas*-Infected Trout Kidney Tissue with the Use of FT-IR Spectroscopy and MALDI Mass Spectrometry Imaging

**DOI:** 10.3390/ijms232012551

**Published:** 2022-10-19

**Authors:** Joanna Matys, Anna Turska-Szewczuk, Barbara Gieroba, Maria Kurzylewska, Agnieszka Pękala-Safińska, Anna Sroka-Bartnicka

**Affiliations:** 1 Department of Biopharmacy, Medical University of Lublin, Chodźki 4a, 20-093 Lublin, Poland; 2 Department of Genetics and Microbiology, Institute of Biological Sciences, Maria Curie-Skłodowska University, Akademicka 19, 20-033 Lublin, Poland; 3 Independent Unit of Spectroscopy and Chemical Imaging, Medical University of Lublin, Chodźki 4a, 20-093 Lublin, Poland; 4 Department of Preclinical Sciences and Infectious Diseases, Poznan University of Life Sciences, Wołyńska 35, 60-637 Poznań, Poland

**Keywords:** trout diseases, Fourier transform infrared spectroscopy, biomolecular profiling, *Aeromonas*, matrix-assisted laser desorption/ionization mass spectrometry imaging, cell membrane disruption, kidney lipids and proteins

## Abstract

*Aeromonas* species are opportunistic bacteria causing a vast spectrum of human diseases, including skin and soft tissue infections, meningitis, endocarditis, peritonitis, gastroenteritis, and finally hemorrhagic septicemia. The aim of our research was to indicate the molecular alterations in proteins and lipids profiles resulting from *Aeromonas sobria* and *A. salmonicida* subsp. *salmonicida* infection in trout kidney tissue samples. We successfully applied FT-IR (Fourier transform infrared) spectroscopy and MALDI-MSI (matrix-assisted laser desorption/ionization mass spectrometry imaging) to monitor changes in the structure and compositions of lipids, secondary conformation of proteins, and provide useful information concerning disease progression. Our findings indicate that the following spectral bands’ absorbance ratios (spectral biomarkers) can be used to discriminate healthy tissue from pathologically altered tissue, for example, lipids (CH_2_/CH_3_), amide I/amide II, amide I/CH_2_ and amide I/CH_3_. Spectral data obtained from 10 single measurements of each specimen indicate numerous abnormalities concerning proteins, lipids, and phospholipids induced by *Aeromonas* infection, suggesting significant disruption of the cell membranes. Moreover, the increase in the content of lysolipids such as lysophosphosphatidylcholine was observed. The results of this study suggest the application of both methods MALDI-MSI and FT-IR as accurate methods for profiling biomolecules and identifying biochemical changes in kidney tissue during the progression of *Aeromonas* infection.

## 1. Introduction

*Aeromonas*, Gram-negative facultative anaerobes, are ubiquitous inhabitants of aquatic and terrestrial ecosystems [1]. They are regarded as opportunistic pathogens of aquatic animals, causing a multitude of infectious diseases in fish [2], reptiles, amphibians [3,4,5,6], and mammals [7]. Several species, including *Aeromonas hydrophila*, *Aeromonas caviae*, *Aeromonas veronii*, are frequent etiologic agents of fish diseases [8] (wherein *A. hydrophila* is considered as predominant pathogen from all motile *Aeromonas*) that are able to under stress provoke motile *Aeromonas* septicemia (MAS) [9] manifested clinically with ulcerations, hemorrhages, abscesses, ascitic fluid and anemia [8], clinical conditions related to systemic infection contributing to high mortality and substantial economic losses to the aquaculture industry worldwide [10], and motile *Aeromonas* infection (MAI) as a chronic disease with symptoms of skin lesions, fin erosion and ulcerations [11]. The non-motile psychrophilic *Aeromonas* species represented by *Aeromonas salmonicida* subsp. *salmonicida* evokes furunculosis, a disease that particularly affects salmon, trout, and char, causing the death of these fish species within a few hours [12]. The pathogenic potential of motile species, mainly *A. hydrophila*, *A. veronii*, *A. caviae*, *A. dhakensis* was associated with a variety of human intestinal and extraintestinal infections, among which bacteraemia, gastroenteritis, and wound infections are the most frequently occurring pathologies [13]. *Aeromonas* have been also documented as being involved in respiratory, hepatobiliary, and urinary tract infections [14]. Moreover, septicemia occurs in susceptible hosts, i.e., immunodeficient or immunoincompetent [15], for instance, those who have leukemia with nonhematologic malignant tumors and hepatobiliary diseases such as hepatitis and liver cirrhosis, and the elderly or infants [16]. *Aeromonas* possess a number of virulence mechanisms that are contributed to the pathogenesis of infection [17,18]. The production of extracellularly secreted enzymes and effector proteins/toxins, such as hemolysins, cytotoxic enterotoxins, proteases, lipases, as well as adhesion molecules (i.e., pili and lateral flagella), capsules, and the ability to biofilms formation, are known to be critical virulence-associated factors in both initial stages and progression of disease [7,19]. As it is commonly known, they are assigned a significant role in the regulation of bacterial virulence and pathogenicity by participating in infecting the host tissues and manipulating the host’s innate and adaptive immune responses [20]. In many epidemiological investigations, it has been shown that the severe course of infection with *A. hydrophila* is related to a mechanism involving many exotoxins, including cytotoxic heat-labile (Alt) and cytotoxic heat-stabile enterotoxins (Ast), cytotoxic heat-labile enterotoxin (Act), *Aeromonas* beta-hemolysin called aerolysin (Aer), cholera toxin-like factor, and other cytolysins and hemolysins that enhance their pathogenicity. *Aeromonas* disease may be complicated by chronic edema or disseminated intravascular coagulation mediated by the release of tissue factors from necrotic tissue into the bloodstream, which is another determinant responsible for the severe course of infection. In motile *Aeromonas* septicemia, hemolysin and aerolysin have been found to be the primary pathogenic factor contributing to the disease [21], but the latest studies demonstrated that four virulence genes, i.e., aerolysin, elastase, hemolysin, and lipase are involved in disease development [9].

The biological activity of the above-mentioned effector proteins/toxins is multidirectional and depending on the mechanism through which they contribute to infection, they can be divided as follows:

### 1.1. Membrane-Disrupting Toxins

The mechanism of action of hemolysins and phospholipases is characterized by pore formation and/or destroying the membranes of the host cells. This virulence factor allows the pathogen to invade the host cell; e.g., aerolysin (pore-forming toxin) [22] and glycerophospholipid:cholesterol acyltransferase (GCAT) protein produced by *Aeromonas* species [23]. Aerolysin toxin protein exported by *A. hydrophila* forms a channel in the host cell membrane after proteolytic removal of a C-terminal propeptide, causing destruction of the membrane permeability barrier and target cell death. The role of aerolysin-related cytotoxic enterotoxin (Act) is the ability to lyse red blood cells, damage tissue culture cell lines, and induce high fluid secretion in intestinal epithelial models [20], as demonstrated in the virulence of *A. hydrophila* [24]. In turn, the activity of phospholipases leads to the degradation cell membrane phospholipids, disrupting the function of the membrane and cell death [25].

### 1.2. Intracellular-Targeting Toxins

Toxins within this group lead to inhibition of protein synthesis, causing cellular death, e.g., Shiga-like toxins (Stx) involved in human and animal diseases, among other important causes of bloody diarrhea and hemolytic uremic syndrome (HUS) [26]. Human isolates of *Aeromonas* possess Shiga toxin genes (*stx1* and *stx2*) highly similar to those of the most virulent *stx* gene variants of Shiga toxin-producing *Escherichia coli* [27]. Shiga toxins are protein toxins containing two parts—one part with enzymatic properties, and the other which binds to the target cell surface. The activity of Shiga toxins inhibits protein synthesis of target cells and also evokes apoptosis [21]. *Aeromonas* exotoxin A (AE) is another important virulence factor, recently identified in a clinical case of necrotizing fasciitis (rapidly progressing fatal skin and muscle tissue lesion) caused by *A. hydrophila* infection. AE belongs to the diphthamide-specific mono-ADP-ribosylating toxin family represented by diphtheria toxin (DT), Pseudomonas exotoxin A (PE), and *Vibrio cholerae* cholix (Chx) that specifically modify the diphthamide residue of eukaryotic elongation factor 2 (eEF2), which leads to the inhibition of protein synthesis and thus death of the host cells [28].

### 1.3. Superantigens

The general mechanism of superantigens is stimulation of excessive activation of immune system cells and release of chemical mediators from immune system cells which result in inflammation, life-threatening fever, and shock. As mentioned earlier, enterotoxin Act is an aerolysin-related pore-forming toxin with hemolytic, cytotoxic, and enterotoxic activities, being the main virulence factor of *Aeromonas hydrophila* [24]. The Act activity includes tissue damage and high secretion in intestinal cells, resulting from the stimulation of a proinflammatory response in host cells [28]. The results suggested that Act upregulates the production of proinflammatory cytokines such as tumor necrosis factor-alpha (TNF-α), interleukin-1 beta (IL-1β), and IL-6 in macrophages [24] and also stimulates transcription of the gene encoding inducible nitric oxide synthase (iNOS). Act leads to induce the production of PGE_2_ coupled to the cyclooxygenase-2 (COX-2) pathway. The production of proinflammatory cytokines and iNOS causes extensive tissue damage in the intestinal loops [24,28].

Therefore, on a molecular level, *Aeromonas* causes infectious diseases, exhibiting various clinical symptoms such as skin infection manifested by dermal lesions or/and external ulcers or more severe systemic infection (septicemia), which are mediated by multiple virulence factors. Bacterial exotoxins, as it is well known, bind to receptors on the host cell’s surface leading to conformational modifications in the membrane receptors and triggering an activating signal or inducing the formation of complexes that alter cell morphology and physiology. Consequently, it induces disorders in signaling pathways and disturbances in the metabolism of lipids. The ultimate effect of the disease progression is pathological changes in the biochemical profile of a specific group of compounds. Depending on the effect on a molecular level of bacterial virulence factors in the host cell, it can be determined which biochemical profile (lipid, protein) is changing [29,30]. Hence, investigating the impact of bacterial infection on the morphology and changes in the profile of tissue biomolecules could provide valuable insight into the pathogenesis of diseases caused by *Aeromonas*. For this reason, the aim of our research was to evaluate pathological changes within the content and molecular structure of proteins, lipids, and phospholipids associated with the progress of *Aeromonas* infection in rainbow trout. This allows for the identification of the degree of tissue damage caused by pathogenic bacteria and will enable a better understanding of the biological mechanisms underlying disease development in the future. Whereas these alterations are related to changes in biochemical composition, which are specific for tissue, chemical, and structural analysis of kidney tissues slices (samples), taken from both apparently healthy rainbow trout and two individuals exhibiting different symptoms of aeromonad infections was performed using FT-IR (Fourier transform infrared) spectroscopy and MALDI-MSI (matrix-assisted laser desorption/ionization mass spectrometry imaging). FT-IR and MALDI are effective, highly sensitive, and powerful techniques that can be used in the study of changes in the chemical composition of biological samples. They have the ability to generate direct information about the (bio)chemical molecular and composition and spatial distribution of compounds in biological and histopathological materials. It is worth emphasizing that specific sample preparation (extraction, mineralization, etc.) and staining is not required. However, the analyzed samples should be cut into thin slices before analysis. They do not require knowledge of the sample a priori. Hence, it is worth pursuing the development each of these techniques as an accurate, efficient, and reliable tool for the diagnosis and identification of animal diseases and/or various abnormalities. Monitoring changes in the structure and compositions of lipids, secondary conformation of proteins as well as spectral bands absorbance ratios can provide useful information concerning disease progression. It should be emphasized, however, that MALDI-MS is destructive to the sample unlike FT-IR spectroscopy [31].

The samples were named NA4905 (control) the healthy fish, NA1905 fish with skin infection (dermal lesions), and NC116005 fish with systemic infection (septicemia).

## 2. Results

**Figure 1:**
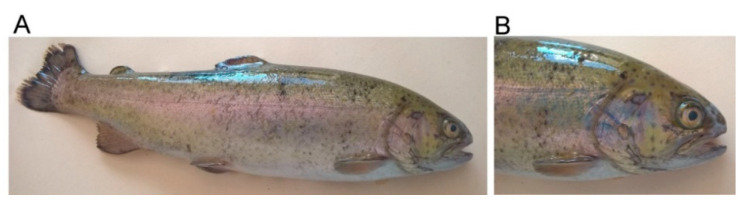
A rainbow trout (*Oncorhynchus mykiss*) naturally infected with *A. sobria*. (**A**) Symptoms of health disorders were manifested as skin infection with visible dermal lesions on the body and erosions on the tail and dorsal fins. (**B**) Dermal lesions were especially found on the trout’s mouth, around the eyes, and along the operculum—the cover of the gills.

**Figure 2:**
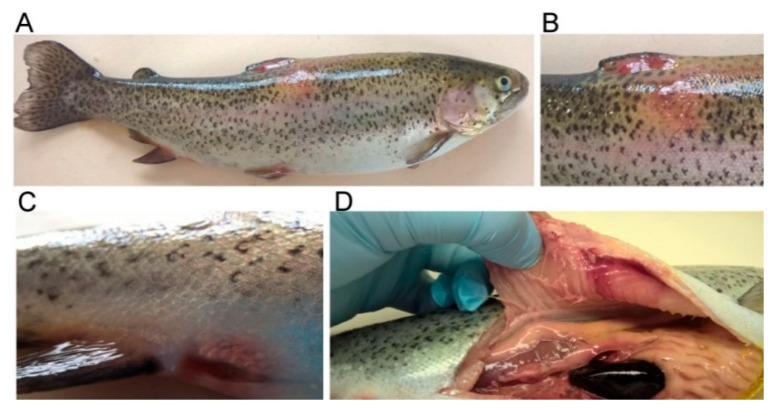
A rainbow trout individual with symptoms of septicemia caused by natural infection with *A. salmonicida* subsp. *salmonicida*. (**A**) In addition to the conspicuous redness and hyperemia of the skin, ruffled scales, swelling and petechias at the base of the dorsal (**B**), ventral, and both pelvic fins, a distended anus (**C**), and a few dermal ulcers, symptoms of systemic infection such as exophthalmia and swelling of abdomen were observed. (**D**) Anemia in internal organs, enlarged spleen and liver, ascitic fluid in peritoneal cavity, and lysis of tissues, the consequence of which was visible as bones sticking out from the muscles.

The bacterial isolates obtained from the tissue samples of the rainbow trout individuals with mild infection (Case 1, NA1905; Figure 1) and systemic infection (septicemia) (Case 2, NC11605; Figure 2) were identified, based on the 16S rRNA gene sequences, as *A. sobria* and *A. salmonicida* subsp. *salmonicida*, respectively. The sequence similarities to those of the reference strains ranged from 99.7% to 100.0%. In all cases, no other bacterial agents were identified. Moreover, no microorganism was identified in the tissues of the healthy trout (control, NA4905).

**Figure 3:**
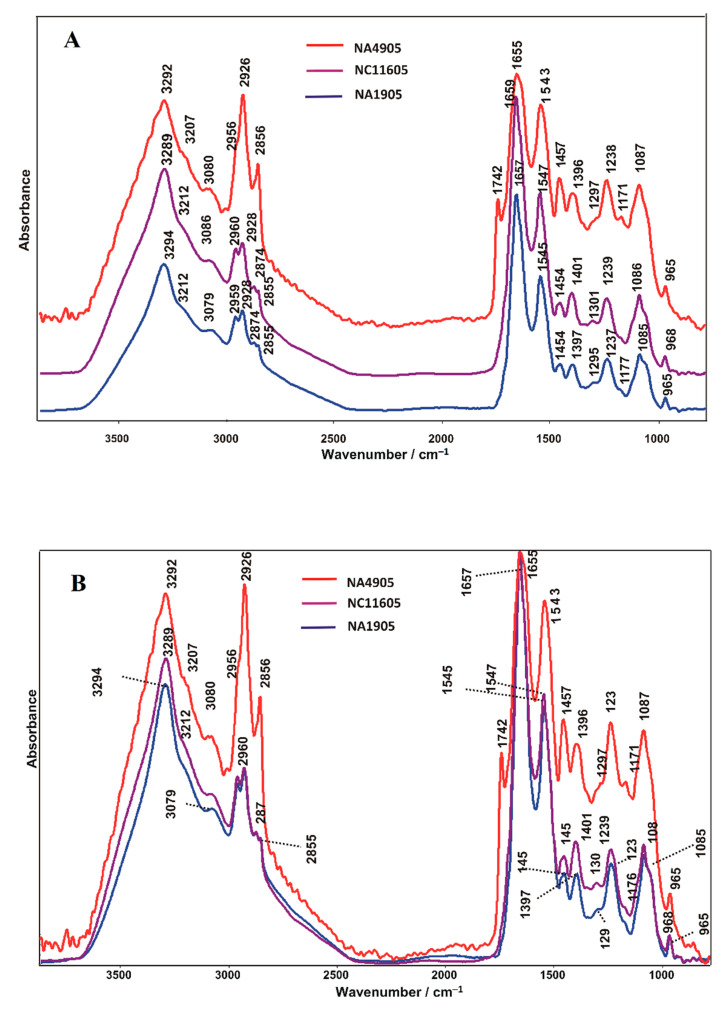
The relative intensity FT-IR spectra of NA4905 (control), NC11605, and NA1905 (pathologically altered rainbow trout kidney tissues) with band assignments—(**A**). FT-IR spectra normalized to the highest intensity band—amide I (**B**).

Figure 3 presents FT-IR spectra of healthy and pathologically altered rainbow trout kidney tissue samples in the range from 3500 to 900 cm^−1^. The main absorption peaks visualized in the mentioned region are dominated by bands related to the absorption modes of proteins and lipids. The characteristic bands obtained in the spectra of NA4905, NA1905, and NC11605 samples and the type of vibrations with assigned tissue components are listed in Table 1.

As can be seen in Figure 3A, there are changes in the intensity of major absorbance bands in the lipid spectral range in NA1905 and NC11605 infected tissues, compared with healthy NA4905 based mainly on the change in the intensity of bands attributed to the asymmetric stretching vibration of CH_3_ (2956 cm^−1^ in NA4905, 2959 cm^−1^ in NA1905, 2960 cm^−1^ in NC11605), asymmetric stretching vibration of CH_2_ (2926 cm^−1^ in NA4905, 2928 cm^−1^ in NA1905 and NC11605), and symmetric stretching vibration of CH_2_ (2856 cm^−1^ in NA4905, 2855 cm^−1^ in NA1905 and NC11605). It may suggest a decreased amount of lipids in both infected tissues. Although the fundamental difference of presented spectra is the disappearance of the phospholipids band at 1745 cm^−1^ assigned to C=O stretching vibration in pathologically altered tissues in contrast to the control, indicating the alteration within the cell membrane. C=O stretching band at ca. 17,405 cm^−1^ can be also attributed to triglycerides, unsaturated cholesterol esters, and free fatty acids. It may as well come from in-phase base C=C and C=O stretching vibrations of DNA. The changes in the intensity of absorbance bands are also observed in the amide spectral range. The contribution of amide I is significantly higher in NA1905 and NC11605 pathological tissue samples than in the case of control.

To facilitate the comparison of individual bands, FT-IR spectra were normalized to the highest intensity band area (to amide I here; this band is usually used for normalization in biological studies)—see Figure 3B. In general, changes in absorbance of lipid bands (in the range 3000–2855 cm^−1^) and protein bands (1657 and 1545 cm^−1^ for amide I and amide II, respectively) are observed. It is clearly visible in the case of bands attributed to asymmetric deformational vibration of CH_3_ and asymmetric deformational vibration of CH_2_ (1457 cm^−1^ in NA4905, 1454 cm^−1^ in NA1905, and 1454 cm^−1^ in NC11605), and symmetric deformational vibration of CH_3_, symmetric deformational vibration of CH_2_ and C=O symmetric stretching of COO^−^ (1396 cm^−1^ in NA4905, 1397 cm^−1^ in NA1905 and 1401 cm^−1^ in NC11605) in proteins and lipids [32]. Additionally, decreased content of lipids was better visualized in pathological tissue samples.

**Figure 4:**
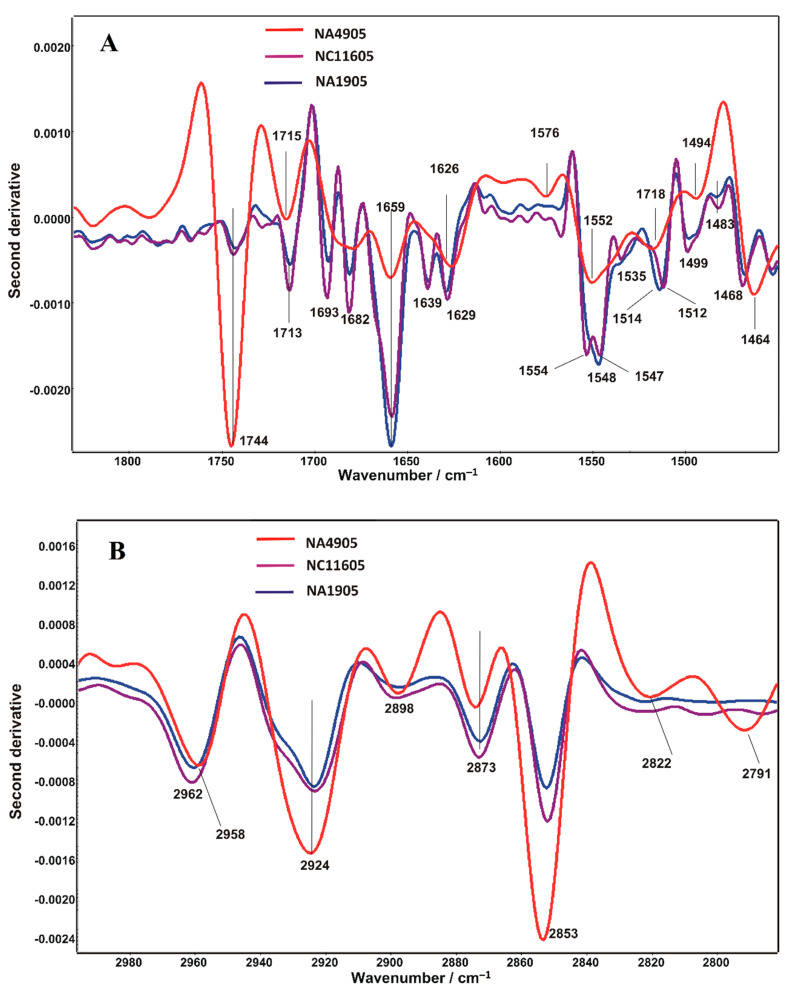
Savitzky–Golay’s second derivative FT-IR spectra in the range from 1800–1450 cm^−1^ (**A**) and 3200–2800 cm^−1^ (**B**).

To analyze the changes in the secondary structure of proteins, the second derivative spectra of samples in the amide spectral range (1800–1450 cm^−1^) was calculated with the Savitzky–Golay algorithm. As can be seen in Figure 4A, there are similar changes in both infected tissues, which clearly differ from the control. The main changes refer to differences in the bands assigned to the β-structures and α-helices (approximately 1750 cm^−1^ and 1650 cm^−1^, respectively).

The disturbances within the cell membrane were also revealed in the lipid spectral range (2800–3000 cm^−1^) of the second derivative course (see Figure 4B). It may indicate an alteration of cell membrane permeability and integrity due to bacterial infection.

**Figure 5:**
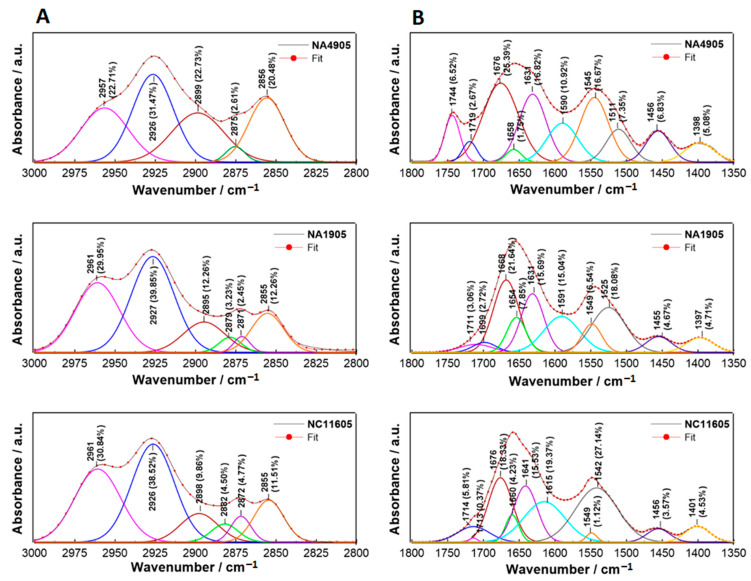
Deconvolution of the lipid (3000–2800 cm^−1^, (**A**)) and protein/lipid (1800–1350 cm^−1^, (**B**)) regions of NA4905, NA1905, and NC11605 samples into separate subcomponents involving Lorentz/Gauss combination peak fitting function. Each band was allocated a maximum and its percentage contribution to the total surface area.

Deconvolution of FT-IR spectra ensures a comprehensive both qualitative and quantitative analysis of conformational changes of lipids and proteins in kidney tissue. Deconvolution of the 3000–2800 cm^−1^ lipid spectral range (dominated by asymmetric and symmetric stretching vibrations of CH, CH_2_, and CH_3_ groups, attributed to alkyl chains primarily present in lipids) [33] and 1800–1350 cm^−1^ mixed protein and lipid spectral range (including phospholipids, amides I and II, asymmetric and symmetric deformational vibrations of CH_2_ and CH_3_ groups in lipids chains) [34] of studied samples is shown in Figure 5.

In the 3000–2800 cm^−1^ range (Figure 3A), deconvolution revealed differences between healthy and infected tissue. In the healthy sample (NA4905), the band assigned to the symmetric stretching vibration of CH_3_ is located at 2875 cm^−1^ and corresponds to 2.61% of the entire bandwidth surface area. In diseased samples, it splits into two bands: 2879 and 2871 cm^−1^ in NA1905, and 2882 and 2872 cm^−1^ in NC11605, while increasing its percentage share compared to the control (summing up 5.68% and 9.27%, respectively). In contrast to the control, in both pathological specimens, a lower percentage of the bands attributed to stretching vibration of CH (2899 cm^−1^ and 22.73% in NA4905, 2895 cm^−1^ and 12.26% in NA1905, 2898 cm^−1^ and 9.86% in NC11605) and symmetric stretching vibration of CH_2_ (2856 cm^−1^ and 20.48% in NA4905, 2855 cm^−1^ and 12.26% in NA1905, 2855 cm^−1^ and 11.51% in NC11605) is observed. In the case of bands assignment to the asymmetric stretching vibration of CH_3_ (2957 cm^−1^ and 22.71% in NA4905, 2961 cm^−1^ and 29.95% in NA1905, 2961 cm^−1^ and 30.84% in NC11605) and CH_2_ (2926 cm^−1^ and 31.47% in NA4905, 2927 cm^−1^ and 39.85% in NA1905, 2926 cm^−1^ and 38.52% in NC11605) groups, the tendency is opposite—their percentage increase. The above-described changes are slightly more pronounced in NC11605 than in the NA1905 sample, indicating more advanced alterations in the structure of cellular membrane lipids. In the sample with dermal lesion NA1905, skin defects are only superficial, while in the sample from fish with septicemia NC11605, there is a much more advanced destruction of cell membranes and tissue barriers, which results in more advanced alterations in the structure of cellular membrane lipids. The bands were assigned on the basis of data included in [35,36].

Deconvolution of the 1800–1350 cm^−1^ range (Figure 5B) allows tracking modifications of the secondary structure of proteins as well as the molecular conformation of lipids and ester lipids (phospholipids). In infected tissues, the band at 1744 cm^−1^ assigned to phospholipids (C=O stretching vibration) disappears, which was already visible in the FT-IR spectra. The C=O functional group is also detected at 1719 cm^−1^ in NA4905, 1711 and 1699 cm^−1^ in NA1905, and 1714 and 1713 cm^−1^ in NC11605 [37]. It may suggest variations in the phospholipid profile of cell membranes. Carbonyl groups may also come from carbohydrates and amino acids; however, the lipid fraction has the largest share. Analyzing the amide I and II (1700–1500 cm^−1^) decomposition, it is possible to trace changes in the secondary structure of proteins. The antiparallel β-sheets are found at 1676 and 1511 cm^−1^ in NA4905, 1668 and 1525 cm^−1^ in NA1905, and 1676 cm^−1^ in NC11605, where there is no band at ~1520 cm^−1^. It is worth emphasizing that at 1670–1660 cm^−1^ in the amide I range, β-sheets may overlap with β-turns. The bands at 1658 and 1545 cm^−1^ in NA4905, 1654 and 1549 cm^−1^ in NA1905, 1660 and 1549, and 1542 cm^−1^ in NC11605, where an additional band appears, are assigned to α-helices. In turn, parallel β-sheets are detected at 1631 cm^−1^ in NA4905 and NA1905, and 1641 cm^−1^ in NC11605. Aggregates are only present in NC11605 at 1615 cm^−1^ [38]. In most pathological NC11605 samples, there is a clear decrease in the content of β-structures, an increase in the amount of α-helices, and the appearance of aggregates in relation to the control. The bands at 1590 and 1591 cm^−1^ in NA4905 and NA1905, respectively are due to the asymmetric stretching vibration of carboxylate associated with deprotonation (RCOO^-^) [39], and is not recorded in NC11605. The 1500–1350 cm^−1^ bandwidth is correlated with deformational vibrations of acyl chains of lipids. The bands at 1456, 1455, and 1456 cm^−1^ are assigned to CH_2_, whereas the bands at 1398, 1397, and 1401 cm^−1^ are ascribed to CH_3_ bending vibration of lipids [40] in NA4905, NA1905, and NC11605, respectively. In the case of infected tissue, their percentage share decreases compared to healthy tissue. The cumulative results of deconvolution are summarized in Table 2.

Recorded FT-IR spectra allow to assess the conformation of proteins, lipids distribution, and changes in the cell membrane caused, among others, by the formation of ion channels and pores [41]. Table 3 compiles the absorbance ratio parameters used in the infrared spectroscopy of animal tissues and related biological characteristics. For each calculated parameter, statistically significant differences were achieved in comparison with the control sample.

The amide I/amide II absorbance index, arising from C=O and N-H stretching vibrational modes of amide groups, can be used to monitor the conformational changes in proteins [43]. This coefficient allows for evaluation of the protein degradation degree, on account of the sensitivity of the amide absorptions to protein structure [44]. An increase in the amide I/amide II ratio in infected samples may be associated with progressive damage to the protein components, which in turn causes disturbances in the secondary structure of proteins. A more detailed qualitative and quantitative study of the secondary structure was performed using the second derivative determination and deconvolution procedure in the amide spectral ranges.

The protein/lipid ratios (here amide I/CH_2_ and amide I/CH_3_) provide data about the biological membrane function, because the changes in the lipid-protein content may influence the membrane symmetry and thickness, and cause the conformational modifications in the membrane receptors and ion channels (pore-forming proteins) [45]. This parameter may constitute a useful indicator for detecting tissue alterations connected with pathologies [46]. An increase in the amide I/lipid ratio taking into account both the absorbance of the methyl (CH_3_) as well methylene (CH_2_) groups was observed either for infected NA1905 and NC11605 samples. It may suggest that infection causes disturbances within the cell membrane in both cases, and probably leads to permeability changes, which might result in membrane discontinuity.

The length of aliphatic chains in lipids can be defined on the basis of the CH_2_/CH_3_ ratio. A decreased CH_2_/CH_3_ index in infected tissues suggests shorter aliphatic chains, and a more densely packed structure with reduced space connecting aromatic clusters compared to the healthy tissue [35]. Moreover, a lower CH_2_/CH_3_ ratio indicates a decreasing lipid saturation effect, associated with diversified both protein and lipid distribution within the cell membrane [36]. This reflects changes in the lipid profile as a result of bacterial infection.

The infrared bands at 1454 cm^−1^ and 1400 cm^−1^, specific for the C-H bending vibrations of lipids and amino acid side chains, are evaluative spectral features for discrimination between normal and pathological tissues [47]. The difference in the 1454/1400 cm^−1^ band ratio also indicates a change in the C-O(H) group vibrations in the cells [48]. Healthy cells usually exhibit a higher value of this band ratio than affected cells, and based on this it is possible to distinguish cells with various growth features [33]. A lower 1454/1400 cm^−1^ ratio in the diseased samples may designate different and immanent energy demands for the metabolism of pathological tissues. Immanent energy demand refers to the faster multiplication of bacterial cells, which use more energy to build cellular structures. It testifies to the progressive disease process and increasing tissue damage.

All the ratios described above clearly indicate numerous abnormalities concerning both proteins and lipids in infected trout kidney tissue, suggesting significant disruption of the cell membranes.

Mass spectrometry (MS), especially matrix-assisted laser desorption/ionization mass spectrometry imaging (MALDI-MSI) and Fourier transform infrared spectroscopy (FT-IR) imaging, offers rapid and simple approaches for lipid [49,50,51] and protein [52,53,54] profiling. Among many techniques, MALDI-MSI allows to detect and reflect the spatial distribution of micro- and macromolecules in both whole organisms and specific tissues [55].

The use of the MALDI-MSI technique allows the precise analysis of metabolites produced under the influence of various stress conditions. Moreover, this technique provides a basis for observation of the dynamics of dislocation of various metabolites in situ in animal or plant or tissues without additional modifications of these compounds and thus reflects the physiological conditions in the organism. Determining the presence of given metabolites at a specific location in the tissue can be crucial in understanding their functions in the body. The analysis can be performed without much a priori information about the analyte [56]. MALDI mass spectrometry imaging can be used in the direct analysis of metabolites in a range from small molecules (<500 Da), e.g., drugs or neurotransmitters, to large proteins (up to 70 kDa) [57].

**Figure 6:**
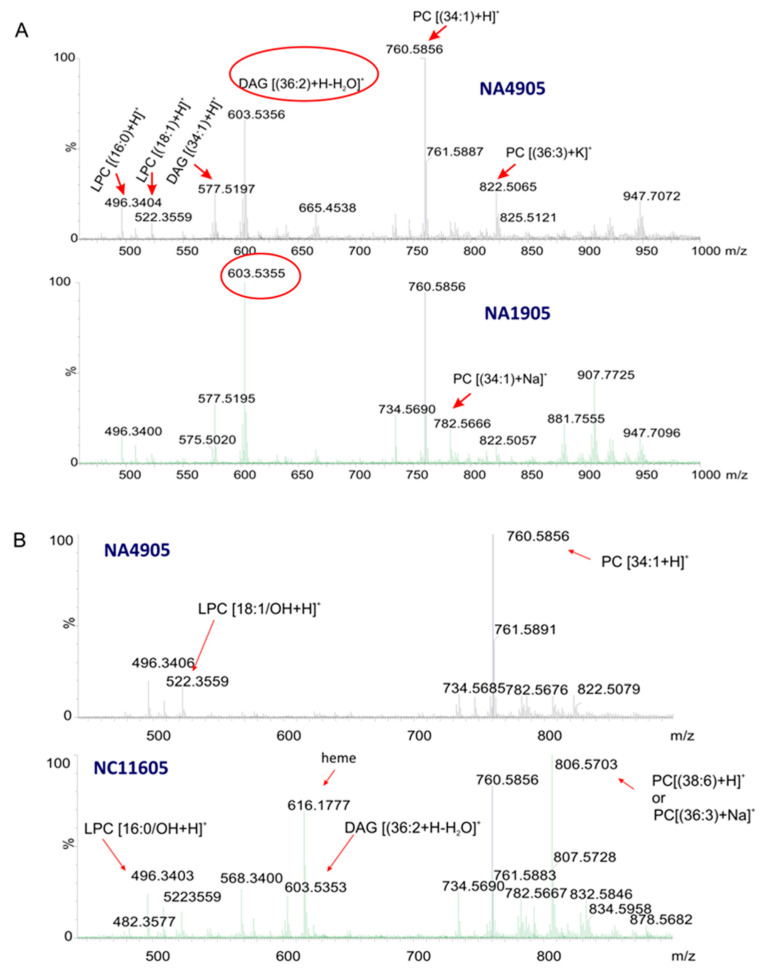
The MALDI mass spectra: (**A**) the average spectrum of ROI at *m/z* 603.5 in the kidney tissue slices of apparently healthy fish (control sample NA4905) and fish with mild infection (dermal lesions) (sample NA1905), (**B**) the average spectrum of ROI at *m/z* 806.5 in the tissue slices of healthy fish (control sample NA4905) and fish with systemic infection (septicemia) (sample NC11605). The red circle in the (**A**) represents the DAG (36:2) peak.

The positive ion mode MALDI-MSI spectrum shown in Figure 6 demonstrates that PC lipids were detected as the predominant ion species, while the peaks corresponding to LPC and glyceryl lipids revealed a lower intensity. No ions assigned to sterols were detected. The ion images of DAG (36:2) at *m/z* 603.53 revealed that phosphocholine and glyceryl lipids ions are dominated. However, these ions appeared to have a different, opposite distribution in tissue slices of healthy and diseased fish (Figure 6).

**Figure 7:**
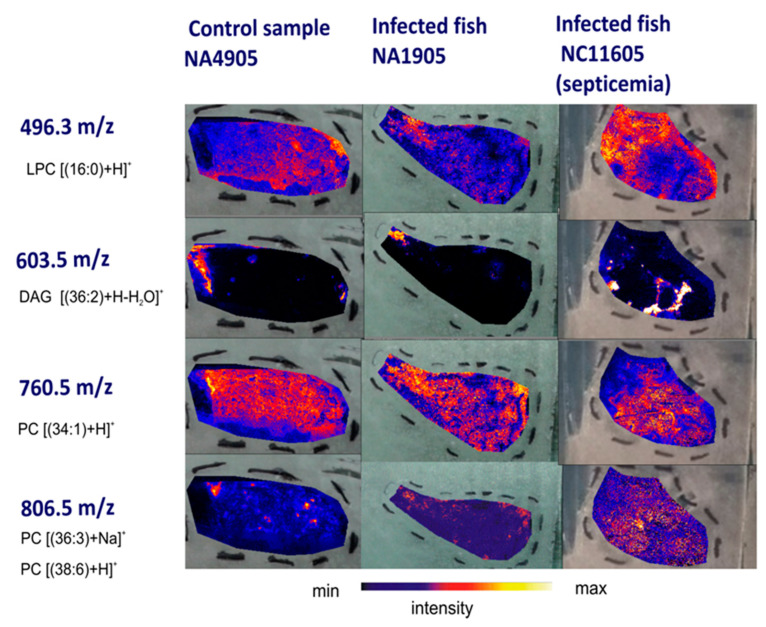
MALDI-MSI images of kidney tissue slices of apparently healthy rainbow trout (control, NA4905) and two other individuals with various clinical symptoms: mild infection (Case 1, NA1905) and systemic infection–septicemia (Case 2, NC11605). The rows present *m/z* of selected lipids with distribution within the samples. The images show distribution of ions in the protonated form [M+H]^+^ at *m/z* 480.3, 760.6, and 603.5, which correspond to LPC (16:0/OH), PC (34:1) and DAG (36:2), respectively. The color bar presents the intensity of compounds.

The ion images of PC (36:3) at *m/z* 806.56 showed that the protonated PC (34:1) ion at *m/z* 760.58 was highly abundant in the tissue slices of trout kidney obtained from healthy fish (Figure 7). The average ROI spectrum of PC (36:3) at *m/z* 806.5 in the tissue sections of diseased fish was dominated by the protonated and sodiated phosphocholine species PC (34:1).

In addition, LPC and glyceryl lipids, and highly abundant ions at *m/z* 616.17 corresponding to the heme prosthetic group of hemoglobin were also detected (Figure 6B).

The heme ion showed the highest intensity in the tissue sections obtained from fish affected by systemic microbial infection (septicemia) following red blood cell and tissue lysis.

## 3. Discussion

*Aeromonas* infection is a result of complex molecular host–microbe interactions, as indicated by effector proteins/toxins secreted into the extracellular medium and/or directly into host cells [58]. Bacterial toxins are virulence determinants that manipulate the functions of host cells and take over the control of vital processes of living organisms to favor bacterial infection [59]. It was found that *Aeromonas* species cause two sorts of cytopathic and intracellular effects. The first type involves rounding of the cells, nuclear condensation, loss of adhesins by the cell layer, and finally cell death. In the second kind, the intracellular effect includes intense cytoplasmic vacuolation with the loss of a well-defined nucleus. The critical to bacterial virulence and interactions with other organisms are protein secretion systems. *Aeromonas* use dedicated various secretion machines, e.g., two-step T2SS, a Sec-dependent system as well as one-step, Sec-independent T3SS and T6SS systems to transport effector proteins/toxins and virulence factors. Type III secretion system (T3SS) is considered the dominant virulence system in *Aeromonas*. The activity of bacterial T3SS effector proteins most often leads to disorders in signaling pathways and reorganization of the cell cytoskeleton [13]. Many authors have focused attention on the biochemical activity of microbial effectors emphasizing their contribution to increased adhesion of pathogen to the host and direct disturbance of target cell function, playing a key role in promoting bacterial virulence [60]. Therefore, monitoring pathological alterations caused by *Aeromonas* infection and determining disease biomarkers can provide valuable insight into the mechanisms of pathogenicity. FT-IR spectroscopy has been increasingly applied as a versatile diagnostic tool in neurodegenerative diseases [61], cardiovascular disorders [62], and cancers [63] by analyzing biofluids, tissues, or cells, which is confirmed in many biological and biomedical studies [64]. Its ability to detect changes in the morphology and chemical composition of intact cells contributed to its utility in discrimination between diseased and normal biological samples [44]. The obtained FT-IR spectra show a molecular “fingerprint” of the whole cell biochemical composition in the examined kidney tissue. Therefore, they represent a unique hallmark of cell lipids, proteins, carbohydrates, and nucleic acid patterns [65]. Due to specific cellular disorders caused by *Aeromonas* infection, in this study, we have focused exclusively on the protein and lipid components.

Monitoring changes in the molecular structure of lipids and secondary conformation of proteins provides useful information to distinguish between normal and infected tissue, thus constituting a crucial aspect in the diagnosis of disease-induced alterations [66]. The deconvolution of the obtained spectra revealed a decreased content of β-sheets in pathological tissue compared to the control; on the other hand, the content of α-helices increased. This result may indicate structural disorders associated with the activity of pore-forming toxins (PFT) secreted by bacteria, used to modulate apoptosis of target cells and cause infection [67]. For example, aerolysin is such a channel-forming toxin and, interestingly, is secreted in an inactive form (as pro-aerolysin) with a β-sheet as the main secondary structure (more than 70% of the molecule) [68]. The transformation of pro-aerolysin to active form takes place only after binding to receptors on the host cell and requires the removal of about 43 amino acids from the C-terminus [69]. Hence, the observed changes in the secondary structure can be indirectly related to the cleavage of pro-aerolysin to active α-toxin, which, due to the ability to form heptameric pores, leads to membrane disorders [70]. This, in turn, degrades the protein components, leading to the disruption of their secondary structures. These results may also suggest the involvement of intracellular-targeting toxins such as Shiga toxins [26] and AE [71] that lead to the inhibition of protein synthesis of target cells [28].

Bacterial proteins target various host proteins involved in cell adherence complexes, metabolite acquisition, molecular transport to the cell membrane, rearrangement of the cytoskeleton, and cell adherence complexes [72]. A particularly useful indicator of protein degradation degree is the amide I/amide II absorbance index [44]. A significant increase in this ratio for pathological tissues, both in NA1905 and NC11605 (see Table 2), may suggest impairment of protein components during *Aeromonas* infection, which may be the result of the activity of effector proteins and toxins secreted by the secretory system, mainly of the third type (T3SS) considered to be the dominant *Aeromonas* virulence system [14,73]. These effectors lead to disturbances in signaling pathways and destabilization of the cellular cytoskeleton, thus contributing to the damage of physiology and phagocytosis [60,74]. Simultaneously, it deactivates the host system alarm that recognizes the infection and induces an immune response [73,74]. The T3SS system facilitates their translocation through the plasma membrane into the host cell or the secretion of pore-forming translocators that facilitate the transport of effector proteins [75,76], and many researchers have indicated its importance and contribution to the multifactorial pathogenicity of *Aeromonas*. It is worth emphasizing that protein effectors secreted by this system are usually critical for bacterial virulence, e.g., loss of T3SS is sufficient to render the bacteria completely avirulent [19,77,78]. AexT is one of the effectors secreted by T3SS causing a detrimental effect on the cytoskeleton of the cell and disrupting actin filaments in the host cells, which may result in progressive damage to protein components. This, in turn, leads to conformational changes in proteins and the rearrangement of proteins [60,73,74]. AexT as a bi-functional toxin contains a GTPase-activating domain and an ADP-ribosylating domain that ADP-ribosylates both muscle and non-muscle actin. Both domains play an independent role in promoting actin depolymerization and cell rounding and are therefore considered highly cytotoxic to host proteins. The AopO (serine/threonine kinase) effector protein activity, in turn, causes changes in the target cell, leading to the disturbance of the normal functioning of actin in the host cell [77], which may also be reflected in the increased amide I/amide II absorbance ratio in tissue samples from diseased fish (see Table 3). It may also be related to enterotoxin Act activity, for which Chopra et al. demonstrated the potential to stimulate the production of proinflammatory cytokines associated with Act-induced tissue damage [24].

Lipids are a major component of the cell membrane, playing a significant role in various cellular processes, including cell signaling and inflammation; therefore, the changes in lipid profile also correlate with disease symptoms, suggesting the importance of lipids as disease biomarkers. Recently, disorders of lipid metabolism have been shown to play an important role in carcinogenesis and development. Moreover, the role of lipids in disease is particularly important in the case of bacterial infections. Lipids act as critical determinants of microbial pathogenesis by altering the structure and functions of the host cell membrane [78]. Membrane phospholipids influence both the structure of the membrane and key metabolic pathways [79]. The organization of the cell membrane monitors cellular functions by modulating the dynamics of the lipid domain as well as biomolecular interactions in the membrane, and therefore depends on the structural properties of the lipids [80]. Since changes in the lipid/protein content can be influenced by the symmetry and thickness of the membrane and lead to conformational modification of membrane receptors and ion channels [45], the protein/lipid absorbance ratio can be used as a biomarker for detecting tissue alterations associated with pathologies providing information about the function of biological membrane.

Bacterial surface ligands, e.g., pathogen-associated molecular patterns (PAMP) and exotoxins, mediate interactions with the host cell during association. It begins with binding to a receptor on the surface of the target cell, which leads to conformational modifications in membrane receptors and causes an activating signal or induces the formation of complexes that disrupt both the physiology and morphology of cells. It causes changes in signaling pathways and leads to disorders of lipid metabolism through the activity of, e.g., GCAT [29]. The amide I/lipid ratio allows the assessment of changes in the integrity and permeability of the cell membrane. Therefore, the increase in the amide I/lipid ratio in both affected tissue samples (NA1905 and NC11605) compared to the normal tissue (NA4905)—see Table 3, may be correlated with the above mechanism and may suggest disturbances in the cell membrane that are likely to cause changes in permeability and discontinuity of membrane. Lipid metabolism disturbances were reflected in decreased intensity bands attributed to deformational vibrations of acyl chains of lipids in the spectral range 1500–1350 cm^−1^ and 2850–2950 cm^−1^, which was confirmed by deconvolution of FT-IR spectra. Moreover, the band assigned to C=O stretching vibration has disappeared in the pathologically altered tissues (see Figure 1), which confirms the variability of the profile of membrane phospholipids.

The MALDI-MSI method was used to demonstrate the distribution of the selected lipids within the samples. The higher intensity of the ion at *m/z* 480.3 in relation to the ion at *m/z* 760.6, resulted from the destruction of lipids, and as a consequence, the increase in the content of the lysolipids such as lysophosphatidylcholine was observed.

These FT-IR spectroscopy and MALDI-MSI methods are well suited for the study of biological samples from all living organisms. They can be applied to study single cells, as well as entire plant and animal tissues [81]. In humans, they can be used as a medical diagnostic tool for many diseases, including cancer, neurodegenerative, bone and joint diseases, diabetes, and cardiomyopathy. It can also be utilized to determine plasma and serum glucose, total protein, urea, triglycerides, chylomicrons, and low-density lipoproteins (LDL) to replace commonly used techniques. In the group of neoplastic diseases, it is applied to distinguish between healthy and pathological tissues, e.g., in the colon, cervix, breast, liver, thyroid, prostate, and brain cancers, and enables the study of body fluids. FT-IR and MALDI techniques enable the detection of changes at an early stage of the disease, impossible to be found by other methods that do not give clear confirmation. Thanks to it, it is possible to determine the degree of malignancy of the neoplasm (in the case of lymphomas) and to monitor the progress of anti-neoplastic treatment, e.g., chemotherapy in the treatment of leukemias and to track the concentration of metabolites formed during therapy. Moreover, it empowers the detection of changes in the secondary structure of proteins and DNA in the course of neoplastic diseases [82].

## 4. Materials and Methods

### 4.1. Fish Samples

Thirty rainbow trout (*Oncorhynchus mykiss*, Walbaum) weighing from 250 to 320 g were randomly collected from every two farms in Poland, according to the OIE procedure (Aquatic Animal Health Code, 2022, https://www.woah.org/en/what-we-do/standards/codes-and-manuals/aquatic-code-online-access/, accessed on 20 September 2022). The purpose of the fish sampling was veterinary examinations performed as part of monitoring studies or to determine the cause of fish health disorders. Additionally, fish farmers approved the use of fish for scientific studies. Live fish kept in water were transported directly to the laboratory within 3 h. The water temperature did not exceed 11 °C.

In all cases, the trout were euthanized by bathing for 5 min, in a solution of an overdose (140 μg L^−1^) of tricaine methanesulfonate (MS-222) (Sigma). Blood from the tail vein was taken immediately after the fish were anesthetized.

Internal organs of both apparently healthy fish (control, NA4905) as well as individuals exhibiting various clinical symptoms (Case 1, NA1905) and Case 2, NC11605) were taken. Symptoms of health disorders were manifested as a skin infection (mild infection) with dermal lesions (NA1905) and systemic infection (septicemia) (NC11605). Samples of tissue were taken aseptically using scalpels for the scrape of the skin disorders and tweezers for internal organs (liver, anterior kidney), according to the OIE procedure (Manual of Diagnostic Tests for Aquatic Animal, 2022, https://www.woah.org/en/what-we-do/standards/codes-and-manuals/aquatic-manual-online-access/, accessed on 20 September 2022).

Skin, liver, kidney, and blood samples of one healthy fish, one individual with skin lesions, and one with systemic infection were taken for routine bacteriological tests. Moreover, the kidney tissues taken from both the healthy fish and the individuals with symptoms of mild or systemic infections were subjected to spectroscopic analyses.

Bacteriological tests samples, after dilution in sterile phosphate-buffered saline (PBS) in a ratio of 1:1 (w/v), were homogenated and inoculated onto agar plates supplemented with 5% horse blood (blood agar, BA) (Biomed, Poland) and onto trypticase soy agar (TSA) (Graso, Poland) and incubated at 25 °C for 48 h.

The dominant types of bacterial colonies were reisolated and identified biochemically as representatives of the genus *Aeromonas* spp. using the API system (bioMérieux, France). However, due to *Aeromonas* species being difficult to distinguish at the species level by conventional biochemical methods, one of the molecular assays was performed [83,84]. The initial characterization of the isolates was confirmed by the polymerase chain reaction (PCR)-amplified 16S rDNA sequencing [85,86].

### 4.2. DNA Extraction

Total genomic DNA was isolated from pure bacterial cultures with the GeneMatrix Tissue and Bacterial Genomic DNA Purification Kit (EURx, Gdansk, Poland) according to the protocol for the isolation of genomic DNA of Gram-negative bacteria. The concentration and quality of DNA were determined using NanoDrop 2000 (Thermo Scientific, Waltham, MA, USA).

The 16S rRNA gene was amplified by PCR using primers 5′-AGAGTTTGATCATGGCTCAG-3′ (forward) and 5′-GGTTACCTTGTTACGACTT-3′ (reverse) according to the procedure described by Borrell et al. (1997) [85]. The PCR reactions were carried out using the Color Opti Taq PCR Master Mix (EURx) according to the manufacturer’s recommendations. The amplicons were checked by electrophoresis in 1% (*w/v*) agarose gel. GeneRuler Perfect^TM^ 100 bp DNA ladder ranging from 100 bp to 2500 bp (EURx) was used as a molecular weight marker. The purified PCR products were commercially sequenced using a 3730xl DNA Analyzer (Genomed S.A., Warsaw, Poland) and aligned with those from the GenBank on the Molecular Evolutionary Genetics Analysis (MEGA, Canterbury, Kent, UK version 7.0) software. Sequences were screened for chimeras using BioEdit sequence alignment editor 7.2. The classification of bacterial isolates to species level was estimated based on a similarity level of ≥99.7% to reference sequences available in the GenBank database. The obtained nucleotide sequences of the 16S rRNA gene (1348 bp and 1409 bp) of *Aeromonas* sp. isolates from the tissue samples were deposited in this database under the following accession numbers: OL778934 (*Aeromonas sobria*) and OL912806 (*Aeromonas salmonicida* subsp. *salmonicida*), respectively.

### 4.3. Sample Preparation for MALDI and FT-IR

The kidney tissues of both healthy fish and individuals exhibiting various clinical symptoms were embedded in Cryomatrix gel, frozen in liquid nitrogen, and stored at −20 °C until spectroscopic examination. The frozen tissue samples were cryosectioned (cut) into 15 micrometers slices using a cryomicrotome (Leica CM 1950, Leica Biosystems, Wetzlar, Germany) and mounted on a microscopic glass (or aluminum- coated glass before FT-IR measurements). Moreover, specimens were stored in the presence of a moisture absorbent (silica gel).

### 4.4. FT-IR Spectroscopy

FT-IR spectra were measured in transflection mode using an FT-IR spectrometer (Nicolet 8700, Thermo, USA) attached to an infrared microscope (Nicolet Continuum, Thermo, USA) with an MCT-A detector. The sample was constantly purged with compressed air during the measurement in order to humidity removal. The microscope was equipped with a ×15 IR objective. Spectra were recorded in the range of 3500–900 cm^−1^ with 120 scans at 8 cm^−1^ spectral resolution. All spectra were measured step-by-step, with a 10 μm step in the x/y plane (linear mode of measurements). For the analysis, ten single spectra were recorded in different points of analyzed samples, averaged into one spectrum representative for a given sample and normalized to the amide I band.

In order to qualitatively determine changes in the molecular structure of lipids and secondary conformation of proteins in the examined samples, second-order derivative spectra were calculated preceded by smoothening using the Savitzky–Golay algorithm (3rd-degree polynomial, 9 points wide sliding window). The spacing between the experimental data was 0.00233506413 cm^−1^. To deconvolute and resolve highly overlapping spectral bands into separate components in the 3000–2800 cm^−1^ and 1800–1350 cm^−1^ ranges, mixed Lorentzian/Gaussian peak fitting was applied. On the basis of the minima of the second derivative, the peaks center for fitting process were determined. Parameters (peak shapes, heights, and widths) were suited to the experimental data utilizing a least-squares interactive procedure. The content of each subcomponent is presented as a percentage, calculated by dividing the area of one band component by the area of the totality of all band component areas present in the studied regions, which allowed the quantitative analysis. The Omnic™ 8 software from Thermo Fisher Scientific (Madison, WI, USA) and GRAMS/AI™ 8.0 software from ThermoGalactic Industries (Waltham, MA, USA) were used to conduct all of the spectral processing.

### 4.5. Statistical Analysis

Absorbance ratios results were obtained from ten spectra and were shown as the mean values ± standard deviation (SD). Statistical analysis was performed by applying an unpaired t-test (Student’s *t*-test), where *p* < 0.05 was considered statistically significant (Statistica 13 software, StatSoft Inc., Mattulsa, OK, USA).

### 4.6. MALDI Mass Spectrometry Imaging

To evaluate the effect of bacterial infection on morphology and changes in the lipids profile of tissue slices obtained from both apparently healthy fish (control NA4905) and individuals exhibiting various clinical symptoms (NA1905 and NC116005), MALDI-MSI has been used. The MALDI-MSI images were measured using a SYNAPT 2G-Si HDMS spectrometer (Waters company). The HDI imaging software was used to process data analysis. A simple desalting protocol using cold 150 mM ammonium acetate was optimized [86]. Washing tissue sections with 150 mM cold ammonium acetate prior to matrix application enhanced signals from protonated PC ions while essentially reducing abundant PC salt adducts. The DHB was used in concentration of 40 mg/mL prepared in TFA:water:methanol (0.1:29:70, *v/v/v*). The images were recorded in the positive ion mode in the mass range of 50–2000 *m/z*.5.

## 5. Conclusions

FT-IR spectroscopic studies showed that *Aeromonas* microbial infection affected important cellular components of the rainbow trout kidney tissue such as lipids, phospholipids, and proteins leading to qualitative and quantitative molecular and structural abnormalities, causing numerous pathological changes. These include:

- increase in the I/amide II absorbance index for infected samples may relate to the conformational changes in proteins resulting from progressive damage to the protein components
- increase in the amide I/lipid ratio (amide I/CH_2_ and amide I/CH_3_) suggests disturbances within the cell membrane in both pathological samples, which probably causes permeability changes and membrane discontinuity
- decrease in the CH_2_/CH_3_ index in infected tissues designates a shorter aliphatic chain and more densely packed structure with limited space linking aromatic clusters in comparison with healthy tissue, and a decreasing lipid saturation effect related to the different distribution of protein and lipids in the cell membrane
- decrease in the 1454/1400 cm^−1^ (C-H bending) ratio in pathologically altered tissues may indicate different and immanent energy demands for the metabolism of the infected sample.

The main contribution of this work is an approach towards the distribution of lipid classes profile within kidney tissue slices as a result of microbial infection. We conclude that the changes in the distribution of ions in the protonated form [M+H]^+^ at *m/z* 480.3, 760.6, and 603.5, which correspond to LPC (16:0/OH), PC (34:1), and DAG (36:2), respectively, provide a reliable lipid profile of tissues under microbial infection. The higher intensity of the ion at *m/z* 480.3 in relation to the ion at *m/z* 760.6 resulted from the destruction of lipids, and as a consequence, the increase in the concentration of the lysolipids, such as lysophosphosphatidylcholine, was detected.

The results of this study suggest the successful application of both cost-effective and time-saving methods MALDI-MSI and FT-IR as accurate techniques for profiling biomolecules and identifying biochemical changes in kidney tissue during the progression of *Aeromonas* infection.

## Figures and Tables

**Table 1:** The most important bands obtained in the FT-IR spectra of tissue samples affected by *Aeromonas* infection.

Wavenumber [cm^−1^]			Vibration Modes *	Tissue Component Assignment
NA4905	Sample NA1905	NC11605		
3292	3294	3289	Stretching (N−H)	Amide A, water
3207	3212	3212	Sym. stretching (N−H)	Proteins
3080	3079	3086	Stretching (N−H)	Amide B
2956	2959	2960	Asym. stretching (CH_3_)	Lipids
2926	2928	2928	Asym. stretching (CH_2_)	Lipids
-	2874	2874	Sym. stretching (CH_3_)	Proteins
2856	2855	2855	Sym. stretching (CH_2_)	Lipids
1742	-	-	stretching (C=O)	Phospholipids
1655	1657	1659	80% stretching CO, 20% stretching CN, bending (HOH)	amide I, water
1543	1545	1547	60% bending (N−H), 30% stretching (C−N), 10% stretching (C−C)	Amide II
1457	1454	1454	Asym. deformational (CH_3_), asym. deformational (CH_2_),	Proteins, lipids
1396	1397	1401	Sym. deformational (CH_3_), sym. deformational (CH_2_), sym. stretching (C=O)	Proteins, lipids
1297	1295	1301	Bending (N−H), stretching (C−N), bending (C=O), stretching (C−C), stretching (CH_3_)	Amide III
1238	1237	1239	Asym. stretching (PO_2_^-^)	DNA, RNA, phosphorylated proteins
1171	1177	1176	Stretching (C−O)	RNA, ribose
1087	1085	1086	Sym. stretching (PO_2_^−^)	DNA, RNA, phosphorylated proteins
965	965	968	Stretching (C−C), stretching (C−O)	DNA, deoxyribose

* sym.—symmetrical, asym.—asymmetrical.

**Table 2:** Collective data showing the results of deconvolution presented in Figure 5.

**Lipid region (3000–2800 cm^−1^)**	**Sample**			**Assignment**
	**NA4905**	**NA1905**	**NC11605**	
	2957 cm^−1^ 22.71%	2961 cm^−1^ 29.95%	2961 cm^−1^ 30.84%	Asym. stretching (CH_3_)
	2926 cm^−1^ 31.47%	2927 cm^−1^ 39.85%	2926 cm^−1^ 38.52%	Asym. stretching (CH_2_)
	2899 cm^−1^ 22.73%	2895 cm^−1^ 12.26%	2898 cm^−1^ 9.86%	Sym. stretching (CH)
	2875 cm^−1^ 2.61%	2879 cm^−1^ 3.23% 2871 cm^−1^ 2.45%	2882 cm^−1^ 4.50% 2872 cm^−1^ 4.77%	Sym. stretching (CH_3_)
	2856 cm^−1^ 20.48%	2855 cm^−1^ 12.26%	2855 cm^−1^ 11.51%	Sym. stretching (CH_2_)
**Protein/lipid region (1800–1350 cm^−1^)**	1744 cm^−1^ 6.52%	-	-	Stretching (C=O)
	1719 cm^−1^ 2.67%	1711 cm^−1^ 3.06%	1714 cm^−1^ 5.81% 1713 cm^−1^ 0.37%	(C=O) functional group
	1676 cm^−1^ 25.39%	1668 cm^−1^ 21.64%	1676 cm^−1^ 18.33%	Antiparallel β-sheets
	1658 cm^−1^ 1.75%	1654 cm^−1^ 7.85%	1660 cm^−1^ 4.23%	α-helices
	1631 cm^−1^ 16.82%	1631 cm^−1^ 15.69%	1641 cm^−1^ 15.53%	Parallel β-sheets
	-	-	1615 cm^−1^ 19.37%	Aggregates
	1590 cm^−1^ 10.92%	1591 cm^−1^ 15.04%	-	Asym. stretching(RCOO^−^)
	1545 cm^−1^ 16.67%	1549 cm^−1^ 6.54%	1549 cm^−1^ 1.12% 1542 cm^−1^ 27.14%	α-helices
	1511 cm^−1^ 7.35%	-	-	Antiparallel β-sheets
	1456 cm^−1^ 6.83%	1455 cm^−1^ 4.67%	1456 cm^−1^ 3.57%	Bending (CH_2_)
	1398 cm^−1^ 5.08%	1397 cm^−1^ 4.71%	1401 cm^−1^ 4.53%	Bending (CH_3_)

**Table 3:** The list of the spectral bands absorbance ratios for particular components of trout kidney samples. The results are presented as mean ± standard deviation (SD).

Biological Indication	The Absorbance Ratio	Sample		
		NA4905 ^1^	NA1905 ^2^	NC11605 ^3^
Protein rearrangement; secondary structure of proteins [36]	Amide I/amide II	1.171 ± 0.128	1.435 ± 0.336 *	1.639 ± 0.303 *
Alteration of cell membrane permeability and integrity [42]	Amide I/CH_2_	2.338 ± 0.406	7.771 ± 0.823 *	9.949 ± 0.622 *
	Amide I/CH_3_	1.906 ± 0.340	3.563 ± 0.689 *	3.545 ± 0.503 *
Lipid saturation [36]; length of lipid aliphatic chains [35]	CH_2_/CH_3_ of lipids	0.817 ± 0.225	0.483 ± 0.149 *	0.361 ± 0.124 *
Metabolism and cell growth rate [33]	1454/1400 cm^−1^ (C-H bending)	1.173 ± 0.087	0.954 ± 0.071 *	0.918 ± 0.069 *

^1^ Amide I 1655 cm^−1^, amide II 1543 cm^−1^, CH_2_ 2856 cm^−1^, CH_3_ 2956 cm^−1^; ^2^ amide I 1657 cm^−1^, amide II 1545 cm^−1^, CH_2_ 2855 cm^−1^, CH_3_ 2959 cm^−1^; ^3^ amide I 1655 cm^−1^, amide II 1543 cm^−1^, CH_2_ 2845 cm^−1^, CH_3_ 2960 cm^−1^; * statistically significant difference (*p* < 0.05) in relation to the control for individual absorbance ratio.

## Data Availability

The data presented in this study are available on request from the corresponding author.
